# SINEUPs are modular antisense long non-coding RNAs that increase synthesis of target proteins in cells

**DOI:** 10.3389/fncel.2015.00174

**Published:** 2015-05-13

**Authors:** Silvia Zucchelli, Francesca Fasolo, Roberta Russo, Laura Cimatti, Laura Patrucco, Hazuki Takahashi, Michael H. Jones, Claudio Santoro, Daniele Sblattero, Diego Cotella, Francesca Persichetti, Piero Carninci, Stefano Gustincich

**Affiliations:** ^1^Scuola Internazionale Superiore di Studi Avanzati, Area of NeuroscienceTrieste, Italy; ^2^Dipartimento di Scienze della Salute, Universita' del Piemonte OrientaleNovara, Italy; ^3^Division of Genomic Technologies, RIKEN Center for Life Science TechnologiesYokohama, Japan; ^4^Cell Guidance SystemsCambridge, UK

**Keywords:** SINEUP, long non-coding RNA, antisense, protein expression, cell lines

## Abstract

Despite recent efforts in discovering novel long non-coding RNAs (lncRNAs) and unveiling their functions in a wide range of biological processes their applications as biotechnological or therapeutic tools are still at their infancy. We have recently shown that AS Uchl1, a natural lncRNA antisense to the Parkinson's disease-associated gene Ubiquitin carboxyl-terminal esterase L1 (Uchl1), is able to increase UchL1 protein synthesis at post-transcriptional level. Its activity requires two RNA elements: an embedded inverted SINEB2 sequence to increase translation and the overlapping region to target its sense mRNA. This functional organization is shared with several mouse lncRNAs antisense to protein coding genes. The potential use of AS Uchl1-derived lncRNAs as enhancers of target mRNA translation remains unexplored. Here we define AS Uchl1 as the representative member of a new functional class of natural and synthetic antisense lncRNAs that activate translation. We named this class of RNAs SINEUPs for their requirement of the inverted SINEB2 sequence to UP-regulate translation in a gene-specific manner. The overlapping region is indicated as the Binding Doman (BD) while the embedded inverted SINEB2 element is the Effector Domain (ED). By swapping BD, synthetic SINEUPs are designed targeting mRNAs of interest. SINEUPs function in an array of cell lines and can be efficiently directed toward N-terminally tagged proteins. Their biological activity is retained in a miniaturized version within the range of small RNAs length. Its modular structure was exploited to successfully design synthetic SINEUPs targeting endogenous Parkinson's disease-associated DJ-1 and proved to be active in different neuronal cell lines. In summary, SINEUPs represent the first scalable tool to increase synthesis of proteins of interest. We propose SINEUPs as reagents for molecular biology experiments, in protein manufacturing as well as in therapy of haploinsufficiencies.

## Introduction

Large genomic projects such as ENCODE (Djebali et al., 2012) and FANTOM (Forrest et al., 2014) have shown that the majority of the mammalian genome is transcribed, thus generating a previously underestimated complexity in gene regulatory networks. Protein encoding genes present a large repertory of alternative Transcription Start Sites (TSSs) that may drive transcription in a cell type-specific manner (Valen et al., 2009). Different 5′UTRs may contain information for mRNA sorting to neuronal compartments as well as for stimulus-dependent translation. Furthermore, in addition to 25000 genes encoding for proteins, at least an equal number of long non-coding RNA (lncRNA) genes have been identified so far. These generate >200 base pairs long transcripts that do not encode for proteins. About one third of annotated lncRNAs overlaps with protein-coding genes (Derrien et al., 2012). Many of these are transcribed from the opposite strand forming sense/antisense (S/AS) pairs (Katayama et al., 2005; Derrien et al., 2012).

The nervous system appears as a privileged site for lncRNA expression, as the vast majority of these transcripts is brain-enriched and regulates neuronal development and functions (Qureshi and Mehler, 2012). Furthermore, a complex network of natural S/AS pairs may participate in brain development and homeostasis in physiological conditions. Interestingly, an increasing number of lncRNAs are associated with brain dysfunction and extensive AS transcription has been measured in *loci* associated to hereditary neurodegenerative diseases (Zucchelli et al., submitted).

Manipulating RNA expression *in vivo* has been proposed as new strategy for molecular therapy. Special attention has been devoted to small antisense oligonucleotides (ASOs) and siRNAs as tools to decrease gene expression of pathological target genes such as, for example, mutant huntingtin in Huntington's disease (Kordasiewicz et al., 2012; Yu et al., 2012).

An equally challenging large group of diseases would strongly benefit from the discovery of RNAs that can increase protein levels of genes for which low expression is pathogenic. Several hereditary intellectual and cognitive disabilities are haploinsufficiencies where only a single functional copy of a gene is unable to produce sufficient protein to maintain a physiological condition (Van Bokhoven, 2011). Therefore an RNA-based drug that can restore physiological amounts of the target protein can in principle be curative. Unfortunately, no molecules that can increase protein levels of a specific mRNA type *in vivo* have been found to date.

In search for AS transcripts that may regulate the expression of Parkinson's disease (PD)-associated genes, we recently discovered AS Uchl1, a natural lncRNA antisense to Ubiquitin carboxyl-terminal esterase L1 (Uchl1/PARK5) (Carrieri et al., 2012). AS Uchl1 is a nuclear-enriched transcript, that is expressed in dopaminergic neurons in the *Substantia Nigra*, the target of PD neurodegeneration, and is down-regulated upon PD-mimicking intoxication *in vitro* and *in vivo* (Carrieri et al., 2015). AS Uchl1 activity increases UchL1 protein synthesis at the post-transcriptional level. Upon stressful insults, AS Uchl1 shuttles from the nucleus to the cytoplasm, where it induces Uchl1 mRNA association to heavy polysomes to increase its translation (Carrieri et al., 2012). AS Uchl1 activity depends on two functional domains: the overlapping region that defines target specificity and the inverted SINE element of B2 subclass (invSINEB2) that confers protein synthesis activation. This functional organization is shared with other lncRNAs part of S/AS pairs in the mouse genome. Finally, by substituting the overlapping region with a sequence antisense to the Green Fluorescent Protein (GFP) mRNA, this synthetic RNA was able to increase GFP protein synthesis with a post-transcriptional mechanism (Carrieri et al., 2012).

The potential scalability of AS Uchl1-derived synthetic lncRNAs to a platform of mRNA-specific translation enhancers remained to be addressed.

Here we propose that AS Uchl1 is the representative member of a new functional class of natural and synthetic RNAs that increase protein synthesis. We name these RNAs as SINEUPs for their activity requires an invSINEB2 element (SINE) to UP-regulate translation of partially overlapping sense mRNAs. The overlapping region is indicated as the Binding Doman (BD) while the embedded inverted SINEB2 element is the Effector Domain (ED). By swapping BD, synthetic SINEUPs are designed targeting mRNAs of interest.

This work shows that synthetic SINEUPs are a versatile tool to increase synthesis of target proteins of interest paving the way for future applications of SINEUPs as molecular biology reagents and for manufacturing of recombinant proteins. Most importantly, SINEUPs may be directed to selected mRNA species *in vivo* representing a new type of RNA-based drug for molecular therapy.

## Materials and methods

### Constructs

Plasmids expressing target proteins were previously described. In particular, for this study we used pEGFP-C2 (Carrieri et al., 2012), pcDNA3-2XFLAG-DJ-1 (Herrera et al., 2007; Zucchelli et al., 2009), pcDNA3-2XFLAG-TTRAP (Zucchelli et al., 2009; Vilotti et al., 2012), pcDNA3-2XFLAG-Hba (Biagioli et al., 2009) (Codrich et al., manuscript in preparation) and pcDNA3-2XFLAG-TRAF6 (Zucchelli et al., 2010, 2011).

Target specific SINEUPs were constructed using pcDNA3-Δ5′-ASUchl1 as backbone (Carrieri et al., 2012). SINEUP-backbone lacks the region of overlap (BD) to Uchl1 and retains AS Uchl1 ED with inverted SINEB2, Alu sequence and 3′ tail. SINEUP target-specific BDs were designed, in antisense orientation, around the ATG of protein-coding sequence with a −40/+32 anatomy.

SINEUP targeting EGFP (AS-GFP, here named SINEUP-GFP) has been described in Carrieri et al. (2012). SINEUP targeting FLAG-tagged proteins (SINEUP-FLAG) was cloned with the following primers (5′ to 3′ orientation):

FWD AS 2xFLAG:

ATATCTCGAGAATTCCTTGTCATCGTCGTCCTTGTAGTCCATCAATTCCAGCACACTGGCGGCCGT

REV AS 2xFLAG:

GAGAGATATCCTCGGATCCACTAGTAACGGCCGCCAGTGTGCTGGAATTGATGGACTACAAGGACG

Primers were annealed, elongated by PCR, digested and ligated into XhoI-EcoRV sites of SINEUP-backbone.

Short SINEUP targeting GFP (miniSINEUP-GFP) was generated combining BD of SINEUP-GFP and ED of AS Uchl1. Briefly, inverted SINEB2 was PCR amplified and cloned into EcoRI and HindIII sites of pcDNA3.1(-). SINEUP-GFP BD was subsequently added to the inverted SINEB2-containing plasmid at XhoI and EcoRI sites to obtain miniSINEUP-GFP. The following primers were used:

FWD EcoRI InvSINEB2: TATAGAATTCCAGTGCTAGAGGAGG

REV HindIII InvSINEB2: GAGAAAGCTTAAGAGACTGGAGC

FWD ApaI all O/L: TATAGGGCCCTCTAGACTCGAG

REV EcoRI O/L GFP20: GAGAGAATTCCAGCACAGTGGCGGCCGC

SINEUPs targeting DJ-1 were generated by annealing and PCR-based method (−40/+32) or with annealing and ligation of phosphorylated oligonucleotides (−40/+4), using the following primers:

SINEUP-DJ-1 (−40/+32) FWD:

ATATCTCGAGGCCAGGATGACCAGAGCTCTTTTGGAAGCCATTTTTATGTTATATGTTT

SINEUP-DJ-1 (−40/+32) REV:

GAGAGATATCTTTCAGCCTGGTGTGGGGCTTGTAAACATATAACATAAAAATGGCTT

SINEUP-DJ-1 (−40/+4) FWD:

TCGAGCCATTTTTATGTTATATGTTTACAAGCCCCACACCAGGCTGAAA

SINEUP-DJ-1 (−40/+4) REV:

TTTCAGCCTGGTGTGGGGCTTGTAAACATATAACATAAAAATGGC

All constructs were verified by sequencing.

### Cell lines and transfection

HEK 293T/17 cells were obtained from ATCC (Cat. No. ATCC-CRL-11268 293T/17) and maintained in culture with Dulbecco's Modified Eagle Medium (GIBCO) supplemented with 10% FBS (SIGMA) and 1% antibiotics (penicillin/streptomycin), as suggested by the vendor. HepG2 cells were kindly provided by Professor Collavin L. from the University of Trieste, Italy (Lunardi et al., 2009). HepG2 and SK-N-SH were cultured in Eagle's minimal essential medium (SIGMA) supplemented with 10% FBS, 1% antibiotics, 1% GlutaMAX and 1% non-essential aminoacids. HeLa cells were grown with DMEM supplemented with 10% FBS and 1% antibiotics as previously described (Angelini et al., 2007). SH-SY5Y cells were maintained in culture as previously described (Zucchelli et al., 2009). BE(2)M17 were grown in 1:1 MEM-Glutamax (GIBCO)/F12 (GIBCO) supplemented with 10% FBS, 1% antibiotics, and 1% non-essential aminoacids.

When required, HEK 293T/17 cells were treated with rapamycin (SIGMA) at 1 μM for 1 h or at 100 nM for 16 h. DNA damage was induced by doxorubicin (SIGMA), at 1 μM for 1 h or 500 nM for 16 h.

HEK 293T/17 cells were transfected with Fugene HD (Roche) and Lipofectamine 2000 (Life Technologies), following manufacture's instruction. HepG2, HeLa, SH-SY5Y, BE(2)M17 and SK-N-SH cells were transfected with Lipofectamine 2000. All cells were transfected with a 1:6 ratio between sense and SINEUP encoding plasmids, maintaining the conditions described for S/AS Uchl1 (Carrieri et al., 2012). In detail, cells were plated in 6-well plates the day before transfection at 60% (for Fugene HD protocol) or 80–90% (for Lipofectamine protocol) confluency. For transfection with Fugene 0.3 μg pEGFP and 1.8 μg SINEUP plasmid were used; for Lipofectamine 2000 0.6 μg pEGFP and 3.4 μg SINEUP. Cells were collected at 24 h (HeLa cells) or 48 h (HEK 293T/17 and HepG2 cells) after transfection and split in two samples for RNA extraction and Western Blot analysis.

For SINEUPs targeting endogenous mRNAs, SINEUP-encoding plasmid was transfected at the highest dose (4 μg) following manufacture's instructions.

Data of RNA and protein levels were obtained from the same transfection in each replica.

### Western blot

For Western blot analysis, cell pellets were directly resuspended in Laemli sample buffer, briefly sonicated, boiled and loaded on poly-acrilamide gels.

Primary antibodies used in this study include anti-GFP rabbit polyclonal antibody (Life Techologies, Cat. No. A6445), used 1:1000, anti-FLAG M2 (SIGMA, Cat. No. 3165), 1:1000, anti-β-actin (SIGMA), 1:5000, and anti-TRAF6 (Abnova), used 1:500. To detect endogenous DJ-1 protein an antibody produced in our laboratory was used (Zucchelli et al., 2009; Foti et al., 2010). For the detection, anti-mouse-HRP or anti-rabbit-HRP (Dako) in combination with ECL (GE Healthcare) was used. Image detection was performed with Alliance LD2-77WL system (Uvitec, Cambridge). Image quantification was done using Adobe Photoshop CS5.

### RNA isolation, reverse transcription and quantitative RT-PCR (qRT-PCR)

Total RNA was extracted from cells using RNeasy Mini Kit (QIAGEN) following manufacturer's instructions. RNA was treated with on-column DNase I (QIAGEN) followed by a second DNase I digestion in solution (Ambion). Two rounds of DNase digestion were required to avoid plasmid DNA contamination in this experimental setting. Single strand cDNA was prepared from 1 μg of purified RNA using the iSCRIPT™ cDNA Synthesis Kit (Bio-Rad) according to manufacturer's instructions. qRT-PCR reaction was performed on diluted cDNA (1:20) using SYBR-Green PCR Master Mix (Applied Biosystem) and an iCycler IQ Real time PCR System (Bio-Rad). Relative expression was calculated with the ΔΔCt method (Schmittgen and Livak, 2008). Oligonucleotide sequences of primers used in this study for GFP and GAPDH (Carrieri et al., 2012), DJ-1 (Foti et al., 2010), TTRAP (Zucchelli et al., 2009), TRAF6 (Zucchelli et al., 2010) and Hba (Biagioli et al., 2009) were previously described.

SINEUP-GFP, SINEUP-FLAG and SINEUP-DJ-1 were detected with primers designed on the 3′ end of AS-Uchl1 (mAS Uchl1 FWD and REV, Primers 3′) (Carrieri et al., 2012). MiniSINEUP-GFP was quantified using the following primers: pTSinvB2 FWD-RT (CAGTGCTAGAGGAGGTCAGAAGA) and pTSinvB2 REV-RT (GGAGCTAAAGAGATGGCTCAGCACTT).

### Cellular fractionation

For fractionation experiments, GFP/SINEUP-GFP were transfected in 10 cm plates at 1:6 ratio using Lipofectamine 2000. Nucleo cytoplasmic fractionation was performed as previously described (Wang et al., 2006). Nucleus and cytoplasmic RNAs were extracted using Trizol reagent (Invitrogen) following manufacturer's instruction. RNA was eluted and treated with DNase I. The purity of the cytoplasmic fractions was confirmed by qRT-PCR on pre-ribosomal RNA using the following primers (Murayama et al., 2008):

FWD 5′-GAACGGTGGTGTGTCGTTC-3′

REV 5′-GCGTCTCGTCTCGTCTCACT-3′

### Statistical analysis

All data are expressed as mean ± standard deviation on *n* ≥ 3 replicas. Statistical analysis was performed using Excel software. Statistically significant differences were assessed by Student's *t*-test. Differences with *p* < 0.05 were considered significant.

## Results

### SINEUPs: definition and design

As shown in Carrieri et al. (2012), AS Uchl1 stimulates translation of partially overlapping sense protein-coding mRNAs with no effects on RNA levels.

Here we propose AS Uchl1 as the representative member of a new functional class of natural and synthetic antisense lncRNAs that activate translation. We named these lncRNAs as SINEUPs since they take advantage of an embedded invSINEB2 element to UP-regulate translation. Therefore SINEUPs can be considered the first example of gene-specific inducers of protein synthesis.

**Figure 1:**
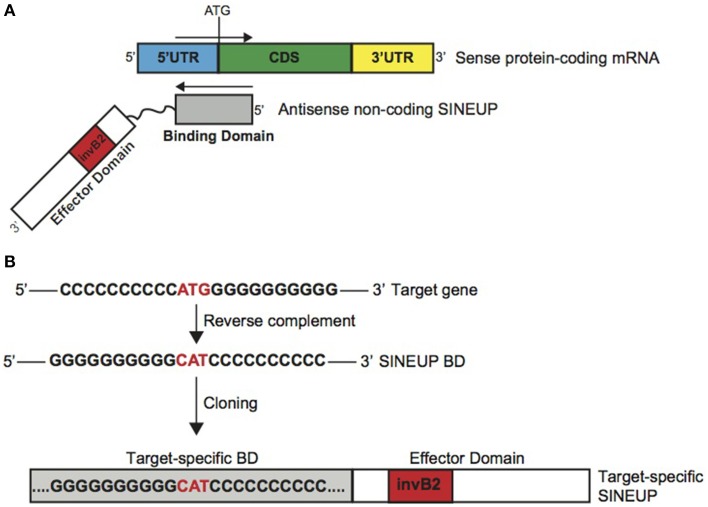
**Schematic representation of SINEUPs. (A)** SINEUP modular structure. SINEUP binding domain (gray): SINEUP sequence that overlaps, in antisense orientation, to the sense protein-coding mRNA. SINEUP effector domain (red): non-overlapping portion of SINEUPs (white), containing the inverted SINEB2 element (invB2) that confers activation of protein synthesis. 5′ to 3′ orientation of sense and antisense RNA molecules is indicated. Structural elements of protein-coding mRNA are shown: 5′ untranslated region (5′UTR, blue), coding sequence (CDS, green) and 3′ untranslated region (3′UTR, yellow). **(B)** SINEUP design strategy. Schematic representation of the cloning strategy to generate target-specific SINEUPs. An artificial target gene sequence is indicated as example. SINEUP domains are represented as in **A**.

SINEUPs display a modular architecture (Figure 1A). In the 5′ region, SINEUPs contain the sequence that overlaps, in antisense orientation, to the sense protein-coding mRNA. We named this sequence as SINEUP's Binding Domain (BD) since it provides target selection and SINEUP specificity by RNA-RNA base pairing. In AS Uchl1, BD is 73 base pair long, centered across the ATG with a −40/+32 configuration, spanning part of Uchl1 5′UTR and a portion of its CDS (Carrieri et al., 2012). The remaining SINEUP sequence presents the embedded invSINEB2 element in the non-overlapping part of the transcript. Since this region has been proven essential for protein synthesis up-regulation, we defined it as Effector Domain (ED) (Figure 1A).

In Carrieri et al., we showed that this modular structure is shared with several natural lncRNAs component of S/AS pair in the mouse genome. For AS Uxt we proved it is able to increase UXT protein synthesis with no effects on Uxt mRNA levels. Therefore these define the first entries in the list of natural SINEUPs.

Given their modular structure, target-specific synthetic SINEUPs can be designed at will by manipulating AS Uchl1 sequence (Figure 1B). In Carrieri et al., we have designed the first synthetic SINEUP directing its activity to GFP mRNA by swapping BDs. However, any invSINEB2 sequence from other natural SINEUPs can in principle sustain activity and be considered potential ED.

Provided with the exact TSS for the target gene, sequence of interest is extrapolated centered across the initiating ATG. After reverse-complement manipulation, gene-specific BD is generated by annealing and PCR amplification of specific oligonucleotides. Target-specific SINEUP is then obtained by cloning specific BDs upstream to the SINEUP effector domain. For expression in mammalian cells, SINEUPs are cloned into pcDNA3.1 plasmid. Vectors for retroviral and lentiviral packaging can also be efficiently used (data not shown).

### SINEUPs work *in vitro* in different cell lines

Mammalian cell cultures *in vitro* are routinely used as model systems to study the molecular mechanisms of gene functions as well as cell factories to produce therapeutic proteins. Considering the flexibility of SINEUP technology and its potential applications in molecular biology experiments, protein manufacturing and therapeutics, we investigated the efficacy and reproducibility of SINEUPs in different cell lines *in vitro*. To this purpose we selected hepatocellular carcinoma HepG2 cells, for their use as a model system by large multicenter consortia, epithelial carcinoma HeLa cells for their wide use in cell biology and in therapeutic protein production as well as HEK 293T/17 cells as positive control of SINEUP activity. As a representative synthetic SINEUP, we took advantage of SINEUP-GFP to increase GFP protein levels in transient overexpression experiments.

We estimated SINEUP activity as fold changes in protein levels encoded by targeted mRNAs in the presence/absence of SINEUP with mRNA amounts kept constant (*p* > 0.05).

**Figure 2:**
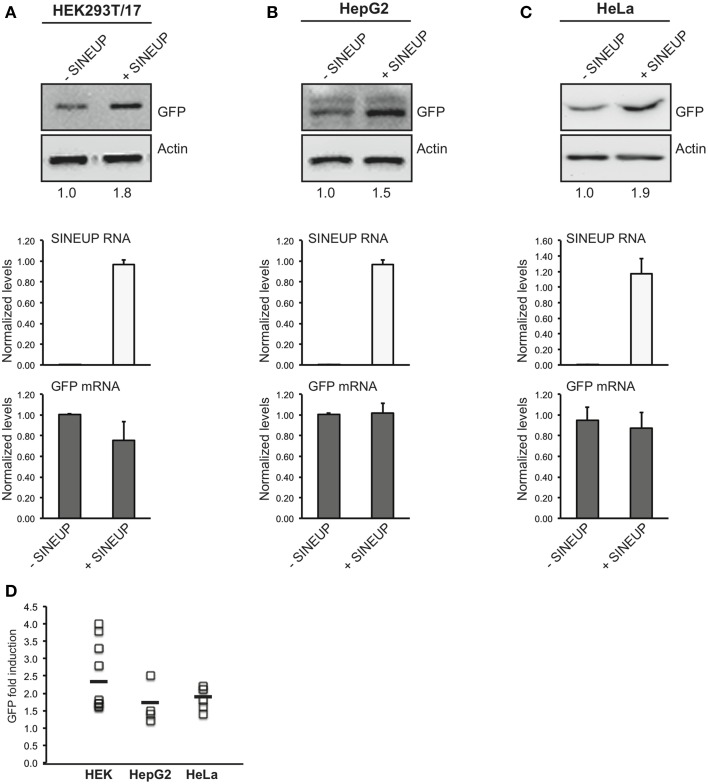
**SINEUP activity in cell lines.** HEK 293T/17 **(A)**, HepG2 **(B)** and HeLa **(C)** cells were transfected with pEGFP-C2 and SINEUP-GFP at 1:6 ratio (+SINEUP). Control cells were transfected with pEGFP-C2 and an empty control plasmid (-SINEUP). 24 h (HeLa) or 48 h (HEK 293T/17 and HepG2) after transfection, cells were lysed and processed for protein (top) and RNA (bottom) levels. Western blot was performed with anti-GFP antibody. β-actin was used as loading control. Fold-induction was calculated on Western blot images normalized to β-actin and relative to empty control samples. Expression of SINEUP-GFP (white bars) and quantity of GFP mRNA (gray bars) were monitored by qRT-PCR using specific primers. Data indicate mean ± standard deviation. Data are representative of >3 independent replicas. **(D)** Graphical representation of SINEUP-mediated GFP up-regulation in HEK (*n* = 10), HepG2 (*n* = 4) and HeLa (*n* = 5) cells. No statistical difference was present between the three cell lines (*p* > 0.05).

Similar fold-changes were observed for SINEUP activity with GFP target at 24 and 48 h after transfection (data not shown). However timing for optimal activity was cell-line dependent, as best conditions were found at 24 h in HeLa and 48 h in HEK 293T/17 and HepG2 cells (Figures 2A–C).

In HEK 293T/17 cells we obtained an average 2.4 fold change (Figure 2A), confirming previously published data (Carrieri et al., 2012) on an independent batch of cells and on a larger cohort of transfections. SINEUP activity ranged from a minimum of 60% induction to a maximum of 400% (Figure 2D). SINEUP effect in transfected HEK 293T/17 cells was not enhanced upon stressful stimuli such as rapamycin and DNA-damage inducing drug doxorubicin (Supplementary Figure 1). HepG2 cells and HeLa cells proved to support SINEUP activity (Figures 2B,C), with an average induction of 1.65 and 1.82-fold, respectively. Minimal values were 20% in HepG2 and 40% in HeLa cells, and top effect was 250 and 220% (Figure 2D). No statistical differences could be measured in SINEUP activity between the three cell lines (*p* > 0.05), albeit HEK 293T/17 cells tended to be more effective (Figure 2D).

We observed that SINEUP activity is maintained independently of the reagent used for transfection. In HEK 293T/17 cells an average of 2.4 fold change could be measured with Lipofectamine (*n* = 5 experiments) and 2.3 with Fugene HD (*n* = 6 experiments) (data not shown). Under these experimental conditions, RNA from transfected SINEUP-GFP was detected in the cytoplasmic fraction although a prominent accumulation in the nucleus was evident (Supplementary Figure 2). Interestingly, sense GFP mRNA was equally distributed between cytoplasmic and nuclear compartments. Altogether these data indicate that SINEUPs can be used *in vitro* in different cell systems to up-regulate proteins of interest.

### SINEUPs can be designed to increase production of target proteins of interest

The modular structure of SINEUPs predicts that by swapping the BD with an appropriate sequence it should be possible to redirect SINEUP activity to target mRNA of interest.

**Figure 3:**
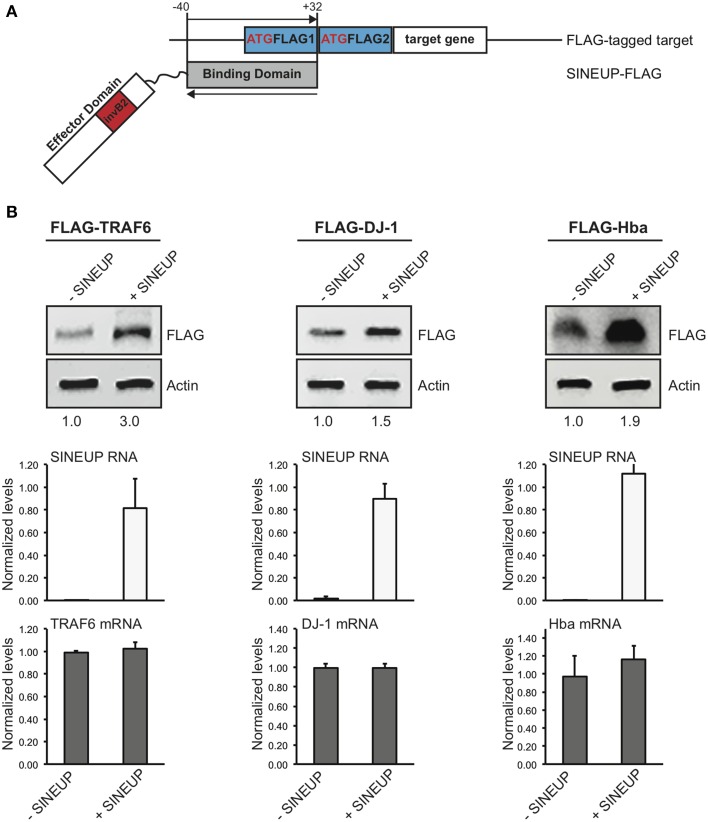
**Examples of target-specific SINEUPs. (A)** Scheme of FLAG tag-specific SINEUP. SINEUP-FLAG was designed to target the −40/+32 region around the ATG of 2XFLAG expression plasmid (pcDNA3-2XFLAG). **(B)** Activity of SINEUP-FLAG was tested in HEK 293T/17 cells transfected with pcDNA3-2XFLAG-TRAF6, DJ-1 and Hba, as indicated. Protein (top) and RNA (bottom) levels were analyzed by Western blot with anti-FLAG antibody and by qRT-PCR with SINEUP (white) and target-specific (gray) primers, respectively. SINEUP activity was calculated as increase in protein levels relative to empty control samples (fold changes are shown). In all conditions, sense RNA quantities were stable (*p* > 0.05). Data indicate mean ± standard deviation and are representative of three independent experiments.

To test the flexibility of BD design, we generated a SINEUP molecule targeting the commonly used FLAG tag sequence. FLAG-specific SINEUP would be able to act at a post-transcriptional level increasing the quantities of proteins expressed in frame with an N-terminal FLAG tag. SINEUP targeting FLAG-tagged proteins (SINEUP-FLAG) was designed to mimic the molecular anatomy of SINEUP-GFP. In particular, SINEUP-FLAG BD encompasses −40 nucleotides (in pcDNA3 plasmid backbone) before FLAG-initiating Met and +32 bases covering the first FLAG tag sequence (Figure 3A).

We took advantage of a series of protein-coding genes available in the laboratory to test SINEUP-FLAG activity. We used human TRAF6 (RefSeq NM_004620) (Zucchelli et al., 2010, 2011), human TTRAP (RefSeq NM_016614) (Zucchelli et al., 2009; Vilotti et al., 2012), human DJ-1 (RefSeq NM_001123377) (Herrera et al., 2007; Zucchelli et al., 2009) and mouse Hba-a1 (RefSeq NM_008218) (Biagioli et al., 2009) (Codrich et al., manuscript in preparation) cloned in pcDNA3-2XFLAG. HEK 293T/17 cells were transfected with plasmids for FLAG-tagged targets in combination with SINEUP-FLAG (+SINEUP). Cells transfected with an empty vector were used as control (-SINEUP). SINEUP activity was measured quantifying protein levels by Western blot and RNA amounts by qRT-PCR. We found that the quantity of three of four FLAG-tagged proteins that we tested was modulated by co-expression of SINEUP-FLAG (Figure 3B). SINEUP effect was different toward the three targets, ranging from 1.5 to 3.0 fold changes. FLAG-TRAF6 showed the strongest SINEUP-mediated activation with a protein induction consistently in the range of 2.6 to 3.0 fold (Figure 3B and data not shown). No effect on TRAF6 mRNA was present, as expected (*p* = 0.46). Interestingly, increased TRAF6 levels could be measured when probing lysates with anti-TRAF6 specific antibody (Supplementary Figure 3).

A 50% up-regulation was measured with FLAG-DJ-1 and 90% with FLAG-Hba. In both cases, sense mRNA in -SINEUP and +SINEUP transfections was not statistically different (*p* = 0.36 in FLAG-DJ-1 samples and *p* = 0.38 in FLAG-Hba).

A modest activation was observed with FLAG-TTRAP where a 10–20% increase was typically observed by Western blot. However, this was consistently accompanied by a similar modulation of TTRAP mRNA levels (Supplementary Figure 4), thus excluding this effect from SINEUP definition.

In summary synthetic SINEUPs can be designed to commonly used tag sequences and the same SINEUP can dictate translation of different tagged proteins of interest.

### MiniSINEUPs containing exclusively BD and ED are active

**Figure 4:**
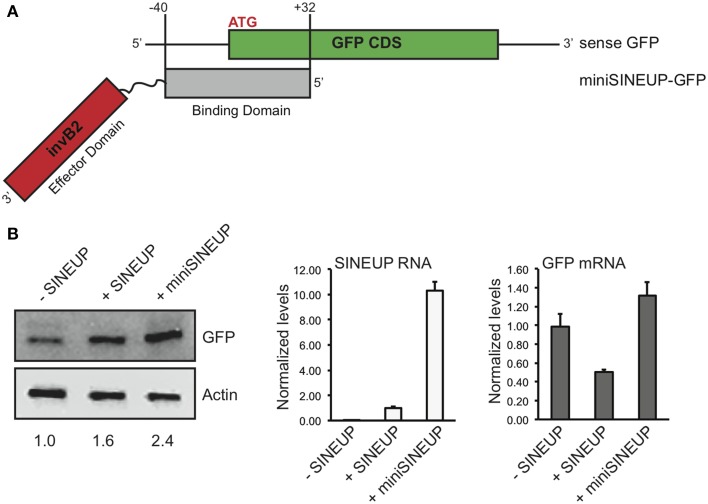
**Short SINEUPs are active. (A)** Domain organization of miniSINEUP-GFP. Binding (gray) and effector (invB2, red) domains are indicated. **(B)** HEK 293T/17 cells were transfected with pEGFP-C2 and miniSINEUP-GFP (+miniSINEUP). SINEUP-GFP was included as positive control (+SINEUP). Control cells received pEGFP-C2 and pcDNA3.1(-) empty plasmid (-SINEUP). Total proteins (left) and RNA (right) were extracted and tested for GFP expression and GFP and SINEUP RNA quantities, respectively. Data are representative of three independent experiments and indicate mean ± standard deviation.

A major limitation in the use of naked RNA for *in vitro* and *in vivo* applications is the instability and low cellular permeability of long molecules. Chemical modifications can by-pass such limits, but with specific constraints in RNA length. Synthetic SINEUPs derived from natural AS Uchl1 are about 1200 nucleotides (nt) long with BD of 72 base pairs, ED of 170 base pairs in addition to intervening sequences, a partial Alu element (73 base pairs) and a 3′ tail. This length is suitable for delivery systems such as viral vectors, but incompatible with the use of SINEUPs as naked RNA therapeutic molecules. Therefore, we aimed at synthesizing the shortest functional SINEUP that retains its translation enhancement activity. MiniSINEUP-GFP was obtained combining SINEUP-GFP BD and ED from AS Uchl1 (Figure 4A) giving rise to a ≈250 nt long transcript. When transfected in HEK 293T/17 cells miniSINEUP-GFP promoted a 2.5 fold increase in GFP protein levels with unaffected mRNA quantities (*p* = 0.11) (Figure 4B). Interestingly, the activity of miniSINEUP was comparable to that obtained with SINEUP-GFP (1.6 fold in this experiment, 2.4 average increase in HEK 293T/17 cells, Figure 2D). Under these conditions, we observed a 10-fold excess of miniSINEUP-GFP RNA relative to canonical SINEUP-GFP, as expected from its reduced size. Despite elevated RNA quantities, no impact was observed on GFP mRNA, proving that miniSINEUP retains the very same post-transcriptional mechanism of its full-length counterpart.

### SINEUPs can be targeted to endogenous mRNAs of interest

The use of SINEUPs as a versatile tool to increase protein synthesis is strictly dependent on their ability to act on endogenous, cellular mRNAs transcribed from a genomic locus that does not present a natural SINEUP antisense gene.

**Figure 5:**
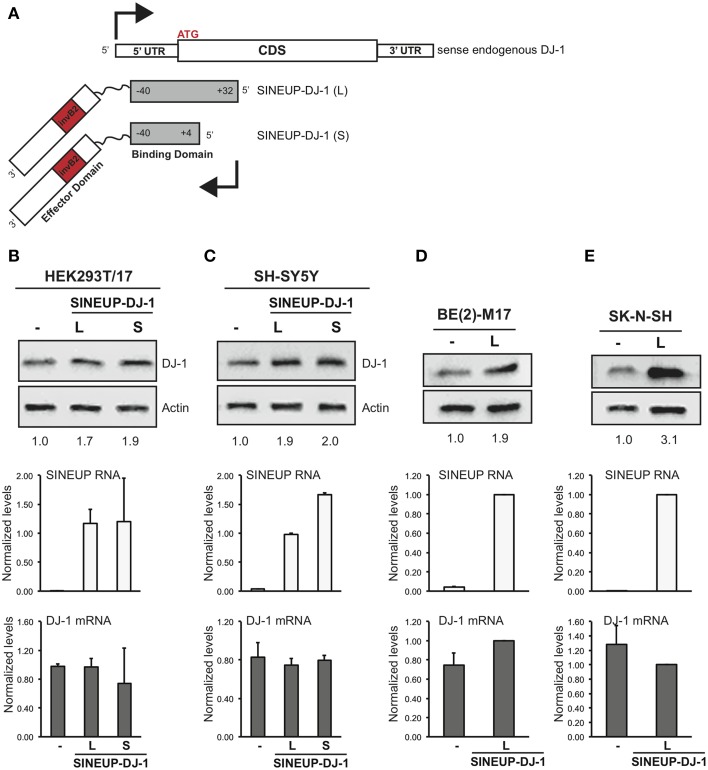
**SINEUP is active on endogenous mRNA. (A)** Scheme of SINEUP-DJ-1. Two different SINEUPs were designed to target the −40/+32 (L) and −40/+4 (S) regions around the ATG of human DJ-1 gene. Activity of SINEUP-DJ-1 was tested in non-neuronal HEK 293T/17 **(B)**, and in neuronal SH-SY5Y **(C)**, BE(2)-M17 **(D)** and SK-N-SH **(E)** cells. Controls were transfected with empty pcDNA3. Protein (top) and RNA (bottom) levels were analyzed by Western blot with anti-DJ-1 antibody and by qRT-PCR with SINEUP (white) and target-specific (gray) primers, respectively. SINEUP activity was calculated as increase in protein levels relative to empty control samples (fold changes are shown). In all conditions, DJ-1 mRNA quantities were stable (*p* > 0.05). Data indicate mean ± standard deviation and are representative of three independent experiments.

To prove this crucial point we designed synthetic SINEUPs targeted to endogenous DJ-1 mRNA, a gene involved in recessive familial Parkinson's Disease (PD). We generated two SINEUP-DJ-1 constructs with two different BD elements: −40/+32 (long, L), from −40 nucleotides (in annotated 5′ untranslated region) before DJ-1 translation start site to +32 bases in the coding sequence, as well as −40/+4 (short, S), ending at the first nucleotide at 3′ of ATG (Figure 5A). Together with HEK 293T/17, we also carried out experiments in three human neuronal cell lines [SH-SY5Y, BE(2)-M17 and SK-N-SH cells] as representative of those frequently used as *in vitro* system to study the function of PD-associated genes. Cells transfected with an empty vector were used as control (-SINEUP).

As shown in Figure 5B, SINEUP activity on endogenous DJ-1 ranged from 1.7 to 2 fold with no changes in endogenous DJ-1 mRNA levels. SINEUP-DJ-1 activity was confirmed in all three human neuronal cell lines, proving the versatility of the technology (Figures 5C–E). Interestingly, the highest induction of DJ-1 protein levels was measured in SK-N-SH cells (3 fold).

In summary synthetic SINEUPs can be designed to mRNAs of interest that do not present a natural SINEUP in their genomic locus opening up the scalable use of SINEUPs to target endogenous protein coding transcripts of mammalian cells.

## Discussion

To our knowledge SINEUPs are the only tool available so far that uses lncRNAs to enhance translation of target proteins. Their modular structure allows synthetic design of an overlapping region (BD) to target proteins of interest, without changing the overall structure of the original lncRNA. Indeed, by extracting solely the target-selecting BD and the ED from the original lncRNA, SINEUPs can be resized to miniSINEUPs retaining their activity.

Over competing technologies, SINEUPs have two major advantages: (1) they modulate translation of target mRNAs without introducing stable genomic changes into target cells; (2) their induction of selected protein is typically in a more physiological range (2-fold) than most conventional gene replacement strategies. These features render SINEUPs (and miniSINEUPs) a potentially interesting tool for a number of applications. First, SINEUPs may be used as reagents for molecular biology. As siRNAs have become an invaluable instrument to inhibit gene expression, many cases exist in which increasing the amount of a specific protein is required. SINEUPs may be designed to a single gene of interest or to a tag that is common to several targets and achieve translation enhancement, thus formally becoming the opposite counterpart of siRNAs. This is especially relevant in the nervous system where regulation of translation has an enormous impact on synaptic plasticity and memory formation. The synthesis of specific proteins requires a fine tuned control at single synapse and spines involving the translation of selective subtypes of mRNAs. The special challenges posed by the anatomo-functional organization of the brain require cellular machinery for controlling mRNAs localization according to the morphology and connectivity of single neuronal cell types. This is achieved at least in part by the use of different TSSs leading to the synthesis of mRNA isoforms with specific 5′UTR containing information for subcellular localization and translation (Baj et al., 2011). SINEUPs can thus induce translation from the mRNA isoform expressed in a defined cellular compartment of a selected neuronal cell type increasing specificity.

Here we show that a synthetic SINEUP against the endogenous mRNA for DJ-1, a gene involved in familial PD, is able to increase its protein synthesis. This is important since, to our knowledge, the mammalian genomic DJ-1 locus does not present a natural SINEUP. This experiment thus proves SINEUPs can potentially act on protein coding transcripts of mammalian cells whether or not they are under an endogenous SINEUP-mediated translational control.

Second, considering their effect on translation, SINEUPs may find applications in protein manufacturing. More than 130 therapeutic proteins are currently in use and many more are under development, including antibodies (Leader et al., 2008). Most production strategies have concentrated efforts in optimizing culture conditions and transcription of recombinant genes leaving room for improvement at post-transcriptional level. Recently, large-scale manufacturing platforms have been developed using transiently transfected cells, mainly CHO and HEK293 (Bandaranayake and Almo, 2014). The data presented here and elsewhere (Cotella et al., submitted) support the feasibility of SINEUPs and their potential to be integrated in existing platforms.

Several aspects regulating SINEUP efficacy with selected targets have still to be elucidated. Here we found that, despite identical sequence in the overlapping region, SINEUP-FLAG failed to up-regulate FLAG-TTRAP, whilst being the most effective with FLAG-TRAF6. Furthermore, the very same BD directed to a single mRNA species can increase protein levels with different efficacies according to the host cellular type. Finally, BD of different lengths can act unlike. In this context the role of the secondary structure of the target mRNAs around AUG remains unclear, although different levels of protein increase may be also accounted by turnover rates specific for each protein or cellular context. While testing synthetic SINEUPs against a large repertory of endogenous mRNAs, we have found that a major cause of apparent lack of activity is due to the wrongful assumption that cells express the Refseq mRNA isoform of the gene. As shown by genome-wide analysis of TSS usage in mammalian cells, the complexity of alternative 5′ ends of mRNAs is staggering. Therefore, when available, we routinely interrogate the FANTOM5 dataset of CAGE libraries (Forrest et al., 2014) of the very same cells used in the experiments taking advantage of the online tool Zenbu (http://fantom.gsc.riken.jp/5/) to identify the correct AUG-surrounding region of the mRNA of interest. Importantly, we observed that high number of cell passages negatively influence SINEUP activity (data not shown). Global CAP-dependent translation is maintained through mTOR activity (Laplante and Sabatini, 2012) and is reduced in the majority of stress conditions (Holcik and Sonenberg, 2005; Sonenberg and Hinnebusch, 2009). We have previously showed that inhibition of mTOR with rapamycin induced an increase in UchL1 protein level dependent on the activity of the natural SINEUP AS Uchl1 (Carrieri et al., 2012). This occurs by triggering shuttling of AS Uchl1 RNA from the nucleus to the cytoplasm and the consequential increased association of Uchl1 mRNA to heavy polysomes for efficient translation. Here we showed that rapamycin and other stressors do not influence the amount of protein increase triggered by synthetic SINEUPs. We may hypothesize that by overexpression we saturate the cytoplasmic content of SINEUP RNA and/or the quantity of target mRNAs that can be associated to heavy polysomes. Further dissection of the cellular pathways that control SINEUP function and identification of SINEUP-binding proteins will provide fundamental insights to improve experimental design and answer these fundamental biological questions.

Finally, lncRNAs represent a new frontier in drug-development. The field of RNA therapeutics has emerged for its great potentials and is set to increase the number of targets beyond initial expectations (Kole et al., 2012). RNA-based drugs have been developed in the past two decades often relying on short non-coding molecules, ASOs or siRNAs, to degrade mRNA or miRNAs of interest. Most recently, lncRNAs appear as therapeutic targets of ASO technology (Modarresi et al., 2012), although the field is still at an early phase. The use of lncRNAs as tools to modulate gene expression is vastly unexplored. This suffers from the limited knowledge of lncRNAs' structure/function relationship and from major obstacles in delivering long RNA molecules. In this context, miniaturization of lncRNAs represents a prerequisite toward applicability in therapeutics. Here we demonstrate that SINEUPs' modular architecture can be employed to construct a miniSINEUP that maintains full-length activity with a length within the small RNA range. Knowing the ED tridimensional structure may provide further insights to help SINEUPs design and optimize activity with minimal length requirements.

In current medical practice there are several unmet therapeutic needs for increasing protein levels *in vivo*. Among them, haploinsufficiency is a condition that arises when the normal phenotype requires the protein product of both alleles, and reduction to 50% or less of gene function results in an abnormal phenotype. This is the cause of a wide spectrum of diseases including ataxias and intellectual and cognitive disabilities. An efficient SINEUP activity specific for the gene of interest would be in principle curative. Furthermore, in many complex and metabolic diseases the increase of pro-survival factors and dysregulated enzymes may impact the well being of patients. As an example, augmented production of neurotrophic factors has been proposed as therapeutic treatments for the majority of neurodegenerative diseases. Therefore SINEUP molecules specific for the transcripts selectively expressed at the site of injury may potentially slow or stop disease progression. Furthermore, it may avoid the unwanted side effects of unregulated expression in the brain that have halted many clinical trials in the past. Our ability to increase endogenous levels of the PD-associated DJ-1 protein represents the first formal prove that synthetic SINEUPs may be a new class of nucleic acid-based drugs.

We are conscious that any potential application of SINEUPs in therapy will strictly depend on their efficient delivery. To this purpose we are currently exploring the preservation of their activity with different delivery systems and chemical modifications.

In summary, here we show first evidences that synthetic SINEUPs may represent a scalable platform for manipulating gene expression of single mRNA species within their physiological range in an array of cell lines.

SINEUPs may thus become a new tool for laboratory experiments, for protein manufacturing and for potential therapeutic intervention *in vivo*.

### Conflict of interest statement

Stefano Gustincich, Piero Carninci, Claudio Santoro, Michael H. Jones and Silvia Zucchelli declare competing financial interests as co-founders and members of TransSINE Technologies (www.transsine.com). Stefano Gustincich, Piero Carninci and Silvia Zucchelli are named inventors in patent issued in the US Patent and Trademark Office on SINEUPs and licensed to TransSINE Technologies. Michael H. Jones is CEO of Cell Guidance Systems, a company distributing SINEUPs as laboratory reagents. Stefano Gustincich and Piero Carninci are co-founders of PARKscreen, an Italian SME aimed to use and develop therapeutic SINEUPs.

## Supplementary material

Supplementary Figure 1 **Treatment with stressful stimuli does not increase SINEUP activity in transfected cells**. HEK 293T/17 cells were transfected with pEGFP in combination with SINEUP-GFP (+SINEUP) or control plasmid (-SINEUP). After transfection, cells were treated with rapamycin or doxorubicin as indicated. Lysates were probed anti-GFP antibody. β-actin was used as loading control. Fold-induction was calculated on Western blot images normalized to β-actin and relative to empty control samples.

Supplementary Figure 2 **SINEUP RNA is detected in the cytoplasm of transfected cells**. HEK 293T/17 cells were transfected with pEGFP in combination with SINEUP-GFP (+SINEUP). RNA was purified from separated nuclear and cytoplasmic fractions. RNA was reverse transcribed and probed for SINEUP RNA and GFP mRNA, as indicated. Purity of nuclear and cytoplasmic fractions was monitored by qRT-PCR on precursor rRNA. Data were normalized to the level of GAPDH in each fraction and analyzed with the ΔΔCt method. RNA levels in the cytoplasm were set to 1. Data indicate mean ± standard deviation and are calculated on 3 independent replicas.

Supplementary Figure 3 **SINEUP-increased targets can be detected with target-specific antibodies**. HEK 293T/17 cells were transfected with pcDNA3-2XFLAG-TRAF6 in combination with SINEUP-FLAG (+SINEUP) or control plasmid (-SINEUP). Lysates were probed anti-TRAF6 antibody.

Supplementary Figure 4 **SINEUP-FLAG does not increase FLAG-TTRAP protein levels**. HEK 293T/17 cells were transfected with pcDNA3-2XFLAG-TTRAP in combination with SINEUP-FLAG (+SINEUP) or control plasmid (-SINEUP). Lysates were probed anti-FLAG antibody. SINEUP RNA and TTRAP mRNA were quantified by qRT-PCR with specific primers. Data indicate average ± stdev and are representative of *n* = 3 independent experiments.

## Abbreviations

lncRNA: long non-coding RNA
AS: antisense
SINE: short interspersed nuclear element
invSINEB2: inverted SINE of B2 subfamily
PD: Parkinson's disease
TRAF6: tumor necrosis factor receptor associated factor 6
TTRAP/TDP2: TRAF and TNF receptor associated protein/tyrosyl-DNA phosphodiesterase 2
Hba: hemoglobin alpha chain.
