# Supplementary figures and images for: SINEUPs are modular antisense long non-coding RNAs that increase synthesis of target proteins in cells
Source: Front Cell Neurosci. 2015 May 13;9:174. doi: 10.3389/fncel.2015.00174 (PMC4429562; doi:10.3389/fncel.2015.00174)

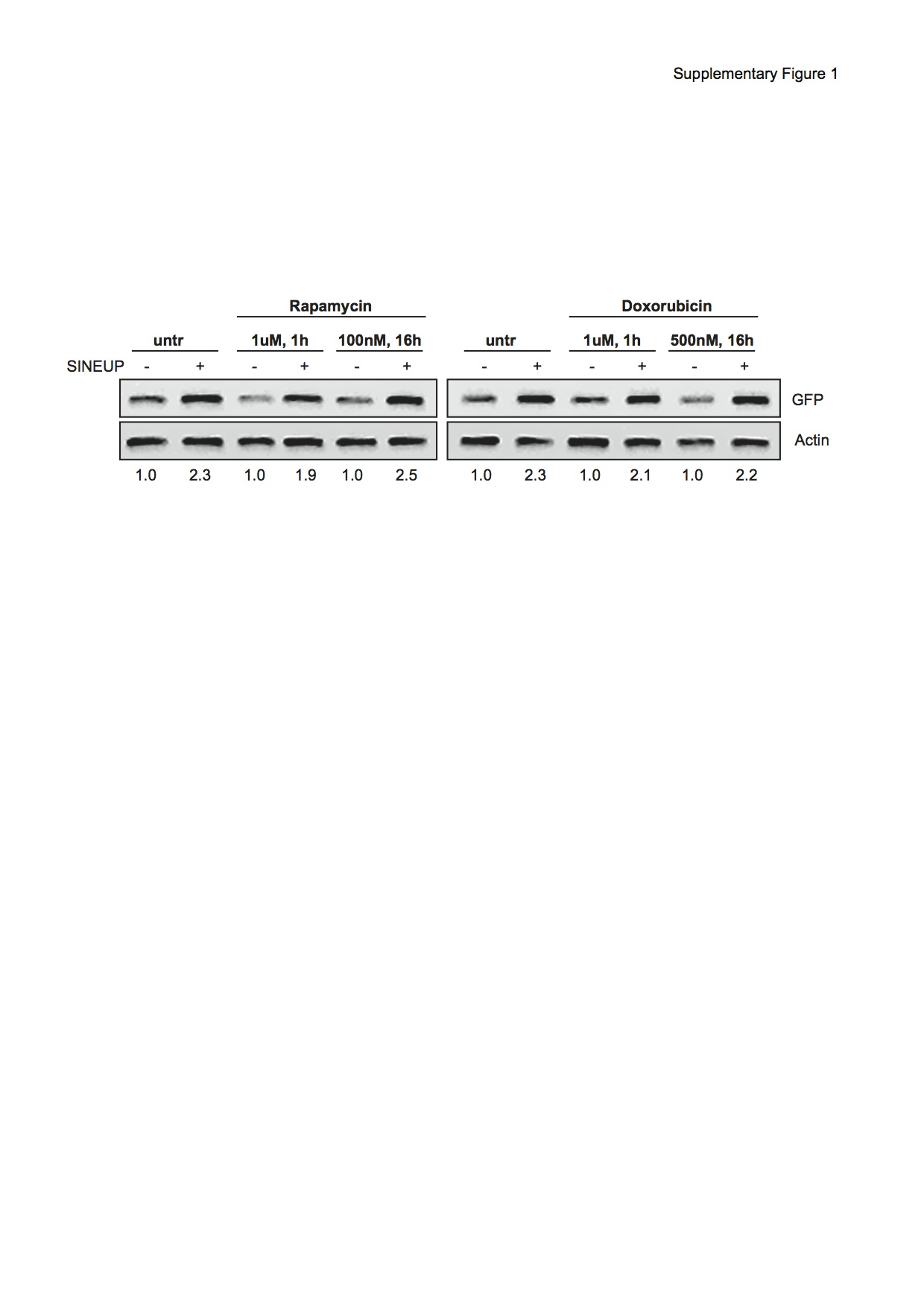

Supplement: Supplementary Figure 1 — Treatment with stressful stimuli does not increase SINEUP activity in transfected cells. HEK 293T/17 cells were transfected with pEGFP in combination with SINEUP-GFP (+SINEUP) or control plasmid (-SINEUP). After transfection, cells were treated with rapamycin or doxorubicin as indicated. Lysates were probed anti-GFP antibody. β-actin was used as loading control. Fold-induction was calculated on Western blot images normalized to β-actin and relative to empty control samples. [file Image1.JPEG]

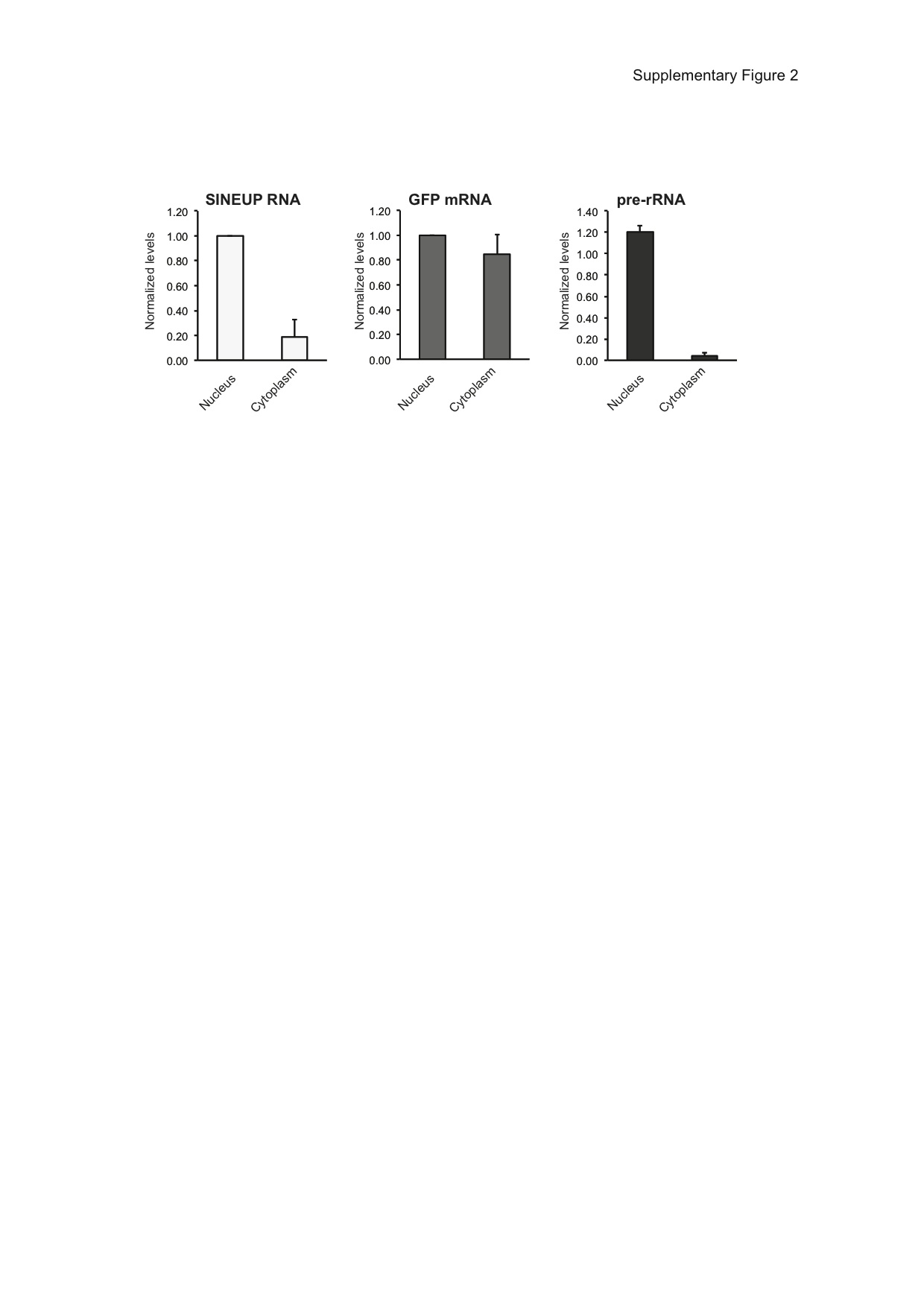

Supplement: Supplementary Figure 2 — SINEUP RNA is detected in the cytoplasm of transfected cells. HEK 293T/17 cells were transfected with pEGFP in combination with SINEUP-GFP (+SINEUP). RNA was purified from separated nuclear and cytoplasmic fractions. RNA was reverse transcribed and probed for SINEUP RNA and GFP mRNA, as indicated. Purity of nuclear and cytoplasmic fractions was monitored by qRT-PCR on precursor rRNA. Data were normalized to the level of GAPDH in each fraction and analyzed with the ΔΔCt method. RNA levels in the cytoplasm were set to 1. Data indicate mean ± standard deviation and are calculated on 3 independent replicas. [file Image2.JPEG]

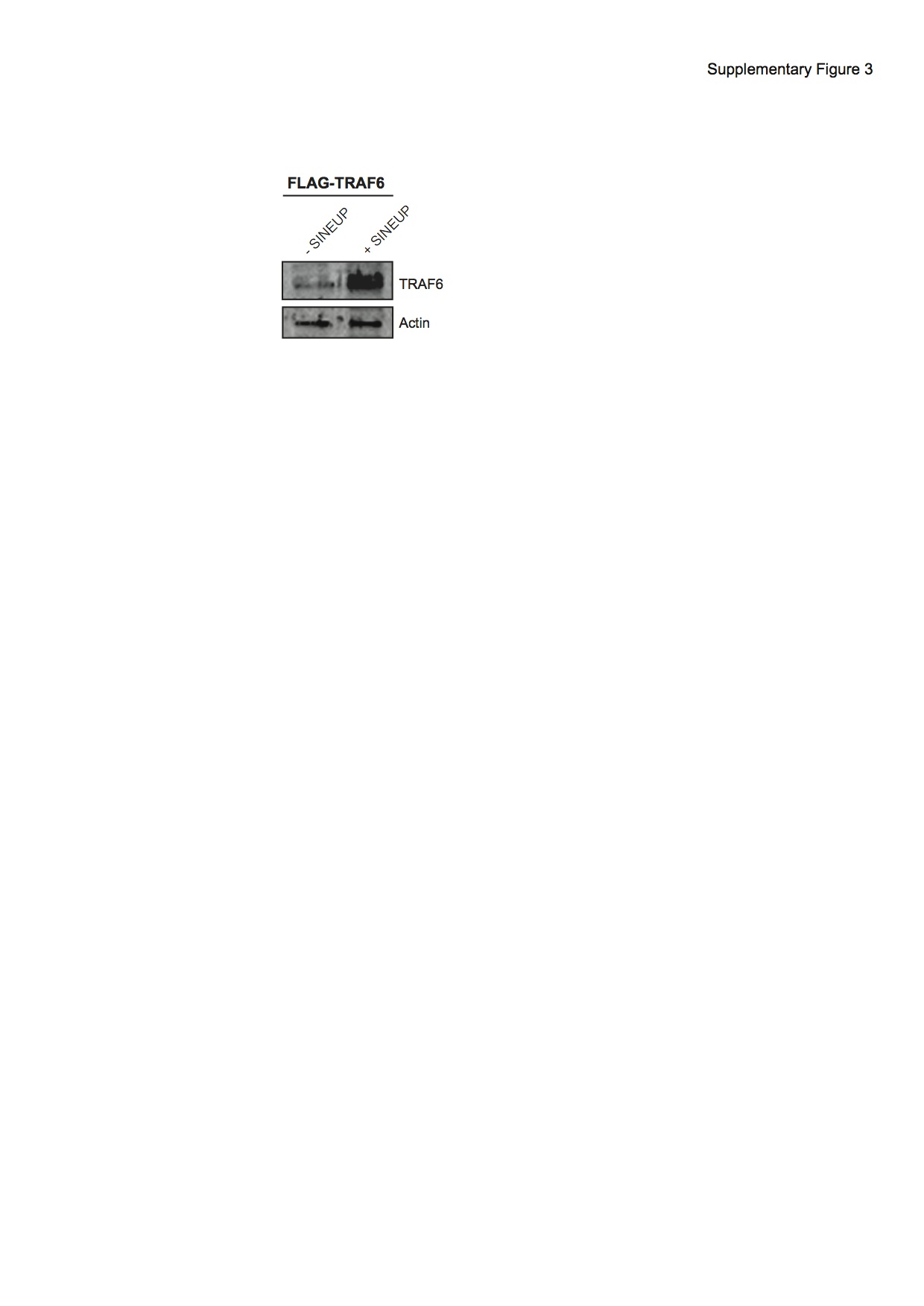

Supplement: Supplementary Figure 3 — SINEUP-increased targets can be detected with target-specific antibodies. HEK 293T/17 cells were transfected with pcDNA3-2XFLAG-TRAF6 in combination with SINEUP-FLAG (+SINEUP) or control plasmid (-SINEUP). Lysates were probed anti-TRAF6 antibody. [file Image3.JPEG]

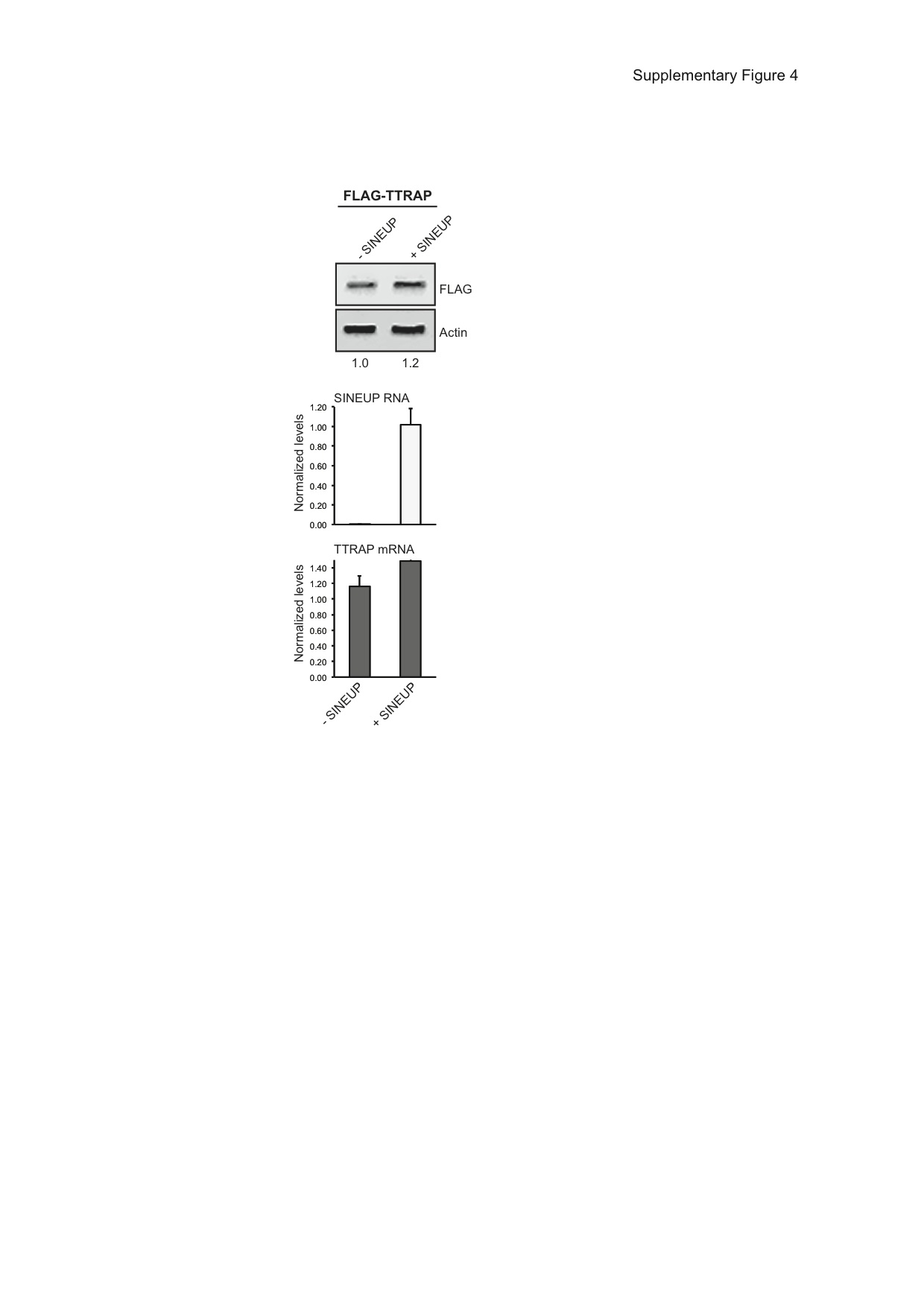

Supplement: Supplementary Figure 4 — SINEUP-FLAG does not increase FLAG-TTRAP protein levels. HEK 293T/17 cells were transfected with pcDNA3-2XFLAG-TTRAP in combination with SINEUP-FLAG (+SINEUP) or control plasmid (-SINEUP). Lysates were probed anti-FLAG antibody. SINEUP RNA and TTRAP mRNA were quantified by qRT-PCR with specific primers. Data indicate average ± stdev and are representative of n = 3 independent experiments. [file Image4.JPEG]
